# Treatment of medulloblastoma with oncolytic measles viruses expressing the angiogenesis inhibitors endostatin and angiostatin

**DOI:** 10.1186/1471-2407-14-206

**Published:** 2014-03-19

**Authors:** Brian Hutzen, Hemant Kumar Bid, Peter J Houghton, Christopher R Pierson, Kimerly Powell, Anna Bratasz, Corey Raffel, Adam W Studebaker

**Affiliations:** 1 The Center for Childhood Cancer and Blood Diseases, The Research Institute at Nationwide Children’s Hospital, Columbus, Ohio 43205, USA; 2 Department of Pathology, The Ohio State University College of Medicine, Columbus, OH 43210, USA; 3 Ohio State University Small Animal Imaging Shared Resource for the Comprehensive Cancer Center and Davis Heart and Lung Institute, Columbus, OH 43210, USA; 4 Department of Neurological Surgery, University of California San Francisco, San Francisco, CA 94143, USA

**Keywords:** Oncolytic measles virus, Angiogenesis, Endostatin, Angiostatin, Medulloblastoma

## Abstract

**Background:**

Medulloblastoma is the most common type of pediatric brain tumor. Although numerous factors influence patient survival rates, more than 30% of all cases will ultimately be refractory to conventional therapies. Current standards of care are also associated with significant morbidities, giving impetus for the development of new treatments. We have previously shown that oncolytic measles virotherapy is effective against medulloblastoma, leading to significant prolongation of survival and even cures in mouse xenograft models of localized and metastatic disease. Because medulloblastomas are known to be highly vascularized tumors, we reasoned that the addition of angiogenesis inhibitors could further enhance the efficacy of oncolytic measles virotherapy. Toward this end, we have engineered an oncolytic measles virus that express a fusion protein of endostatin and angiostatin, two endogenous and potent inhibitors of angiogenesis.

**Methods:**

Oncolytic measles viruses encoding human and mouse variants of a secretable endostatin/angiostatin fusion protein were designed and rescued according to established protocols. These viruses, known as MV-hE:A and MV-mE:A respectively, were then evaluated for their anti-angiogenic potential and efficacy against medulloblastoma cell lines and orthotopic mouse models of localized disease.

**Results:**

Medulloblastoma cells infected by MV-E:A readily secrete endostatin and angiostatin prior to lysis. The inclusion of the endostatin/angiostatin gene did not negatively impact the measles virus’ cytotoxicity against medulloblastoma cells or alter its growth kinetics. Conditioned media obtained from these infected cells was capable of inhibiting multiple angiogenic factors *in vitro,* significantly reducing endothelial cell tube formation, viability and migration compared to conditioned media derived from cells infected by a control measles virus. Mice that were given a single intratumoral injection of MV-E:A likewise showed reduced numbers of tumor-associated blood vessels and a trend for increased survival compared to mice treated with the control virus.

**Conclusions:**

These data suggest that oncolytic measles viruses encoding anti-angiogenic proteins may have therapeutic benefit against medulloblastoma and support ongoing efforts to target angiogenesis in medulloblastoma.

## Background

Medulloblastoma is the most common malignant brain tumor in children, accounting for 20% of all pediatric tumors of the central nervous system [1,2]. Treatment strategies vary according to a system of risk stratification, but typically include surgical resection followed by craniospinal irradiation and chemotherapy [1]. Despite significant increases in overall survival, approximately one-third of medulloblastoma patients will remain refractory to current therapies [3]. Moreover, the majority of survivors will suffer severe and often permanent side-effects such as neurological and cognitive impairment, endocrine abnormalities, and physical disabilities [4,5]. As such, there is a great need for safer and more effective therapies to treat medulloblastoma.

Oncolytic virotherapy may represent such an approach. An oncolytic virus is one that selectively infects and kills neoplastic tissue, leaving the normal surrounding tissue unharmed as it continues to replicate in and lyse transformed cells [6]. We have recently reported on the potential of a recombinant oncolytic measles virus (MV) against medulloblastoma, demonstrating its efficacy in orthotopic mouse models of localized and disseminated disease [7,8]. In each study, intratumoral administration of MV led to tumor stabilization or remission and effectively doubled the median survival times of treated mice. The oncolytic MVs utilized in these studies were based on the highly attenuated Edmonston vaccine strain [9]. Genetically modified derivatives of Edmonston MV are currently being tested in phase I clinical trials against both solid and blood cancers and have thus far been shown to be safe and reasonably effective [10-12]. Although data from these trials is still forthcoming, efforts to develop a new class of oncolytic MVs with enhanced antitumor properties continue to be made and tested in various preclinical models [13,14].

Angiogenesis, the process by which new blood vessels arise from the pre-existing vasculature, is critical for the maintenance and progression of medulloblastoma [15-18]. Therapies that target angiogenic factors might thus be a useful component in the treatment of the disease. Two of the most studied inhibitors of angiogenesis are the endogenous proteins endostatin and agiostatin [19,20]. Endostatin is a naturally occurring fragment of collagen XVIII known to modulate angiogenesis regulatory genes across more than 12% of the human genome [21]. Despite such wide-ranging effects, endostatin exhibits virtually no toxicity and there are no reports of tumors developing endostatin resistance [22]. Angiostatin, a proteolytic cleavage product of plasminogen, inhibits endothelial cell migration and proliferation by interacting with endothelial cell surface proteins such as ATP synthase and angiomotin [23]. Although there is still considerable uncertainty regarding its mechanisms of action, angiostatin has no associated toxicities and has been shown to act synergistically with endostatin [24]. Clinical success has largely eluded endostatin and angiostatin-based therapies however, due to issues such as manufacturing difficulties and short serum half-lives [25,26]. One potential solution to address these shortcomings is the use of gene transfer strategies to systemically deliver a continuous source of endostatin and angiostatin to tumor [27]. To investigate this possibility within the context of oncolytic measles virotherapy, we have developed recombinant MVs that express endostatin:angiostatin (E:A) fusion proteins. Because Edmonston strain MVs are inherently tumor-selective and retain their ability to replicate, an E:A armed MV could potentially result in the targeted inhibition of angiogenesis within the local tumor environment in addition to virus-mediated oncolysis. In this study, we report on the novel construction and characterization of recombinant MVs expressing angiogenesis inhibitors. Furthermore, we demonstrate that oncolytic MVs armed with the angiogenesis inhibitors E:A can induce infected medulloblastoma tumor cells to secrete the angiogenesis inhibitors endostatin and angiostatin without attenuating the oncolytic activity of the MV itself. In addition, the E:A secreted by these infected tumor cells is biologically active and is capable of inhibiting multiple regulators of angiogenesis *in vitro* and *in vivo*.

## Methods

### Cell culture

The 293 T, Vero, D283med, human umbilical vein endothelial cells (HUVEC) and bEnd.3 cell lines were obtained from the American Type Culture Collection. The D425med cell line was obtained from Darrell Bigner (Duke University, Durham, NC). The D283med-luc and D425med-luc cell lines were generated as described previously [7,8]. The 293 T, Vero, D283med, D425med and bEnd.3cell lines were maintained in DMEM supplemented with 10-20% FBS, 1% penicillin/streptomycin and 2 mM L-glutamine and cultured at 37°C in a humidified incubator set at 5% CO_2_. Low passage HUVEC cells were maintained in endothelial cell growth medium M200 (Invitrogen) in high glucose supplemented medium with 10% FBS, endothelial cell growth supplements (Cascade Biologics Inc., Portland Oregon), and 2 mM L-glutamine at 37°C with 5% CO_2_.

### Measles virus plasmid construction and rescue

Plasmids pBLAST-hEndo:Angio and pBLAST-mEndo:Angio were obtained from InvivoGen (San Diego, CA). Amplicons of plasmid DNA encompassing the human Interleukin-2 (IL-2) signaling peptide and the full length endostatin:angiostatin fusion genes were generated using Easy-A high-fidelity PCR cloning enzyme (Agilent Technologies, Wilmington, DE) and the following sets of PCR primers: hE:A forward - 5′CAGCCCATCAACGCGTTAATGTACAGGATGCAACTCCTGTC 3′, hE:A reverse- 5′TAGTATCATCGCGAGACGTCCATGTCATACAACACTCGCTTCTGTTC 3′ and mE:A forward – 5′TAACGCGTACCATGTACAGGATGCAACTC 3′, mE:A reverse – 5′TAGACGTCCTAACTCCCTCCTGTCTC 3′. These PCR products were then cloned into a previously mluI/AatII digested MV-NIS backbone (obtained from Stephen Russell, Mayo Clinic, Rochester, MN) using the InFusion HD cloning system (Clontech, Mountain View, CA) to create plasmids pMV-hEndo:Angio and pMV-mEndo:Angio. The pMV-GFP plasmid and corresponding virus was created by PCR amplifying eGFP from the p(+)MVeGFP plasmid [28] using the following primers: GFP2 forward: 5′CAGCCCATCAACGCGTACGCCACCATGGTGAGCAAG 3′ and GFP2 reverse: 5′TAGTATCATCGCGAGACGTCCAGTCTACTTGTACAGCTCGTCC 3′. The resulting PCR product was cloned into TOPO-pCR 2.1 using the TOPO TA cloning kit (Invitrogen, Carlsbad, CA). The eGFP gene was excised from this plasmid by restriction digestion with mluI and AatII, gel purified, and then ligated into an mluI/AatII opened pMV-hEndo:Angio plasmid to create pMV-GFP2.

Four μg of these pMV plasmids were transfected into 60% confluent 293 T cells alongside the MV accessory plasmids pCA-MVN, pCA-MVP, pCA-MV-L and the T7 polymerase encoding pCA-T7pol (kind gifts of Urs Schneider, University of Freiburg, Freiburg, Germany) [29] using the calcium phosphate method. The media on the transfected cells was changed with fresh DMEM after 24 hours. After an additional 24–48 hours, the transfected 293 T were scraped into their media and overlaid onto 70% confluent Vero cells in 10 cm plates. These cells were then incubated at 37°C over the next several days and monitored periodically for the appearance of syncytia. Once identified, these cells were split and evenly distributed on new plates of 70% confluent Vero cells. After 48–72 hours, the media was removed and the cells were scraped into a minimal volume of OptiMEM (Invitrogen, Carlsbad, CA). The collected cells were then subjected to two cycles of freeze-thawing, followed by centrifugation at 10,000× g to pellet and remove cellular debris. These initial MV products were stored at −80°C and titered the following day as described below.

### MV propagation and titering

MV stocks were propagated by infecting Vero cells at an MOI of 0.01 in a minimal volume of OptiMEM for 2 hours. Unbound virus was then removed and replaced with DMEM with 10% FBS and the cells were incubated an additional 48–72 hours at 37°C. When the majority of the Vero cells had fused into syncytia, the media was removed and the cells were scraped into a small volume of OptiMEM. MV was harvested by two cycles of freezing in liquid nitrogen and thawing, followed by centrifugation at 10,000× g to pellet and remove cellular debris. Aliquoted virus was stored at −80°C. Viral titers were determined by 50% tissue culture infective dose (TCID_50_) titration on Vero cells [30].

### In vitro kill curves

D283med and D425med cells were seeded in 96-well plates at a density of 3x10^4^ cells/well in a volume of 75 μl DMEM. The cells were infected with MOI 0.1 MV after 24 hours of incubation, when they had reached approximately 70-80% confluency. Cell viability was determined using the MTT assay (ATCC, Manassas, VA). Absorbance at 570 nm was measured for each well using a SpectraMax M2 microplate reader (Molecular Devices, Sunnyvale, CA) and compared to an uninfected control at each corresponding timepoint. Each sample and control was run in quintuplicate. The average absorbance for each sample is presented as a percentage of the uninfected controls. Error bars represent +/− one standard deviation.

### In vitro virus production assays

D283med (7.5x10^5^ cells/well) and D425med cells (1x10^6^ cells/well) were seeded in 6-well plates and infected the following day with MOI 0.1 MV in 500 μl OptiMEM. Unabsorbed virus was removed after two hours and replaced with 3 ml fresh DMEM. The cells were scraped into 125 μl OptiMEM at 24, 48 or 72 hours after infection, freeze-thawed twice, and centrifuged. The collected MV was then titered on Vero cells using the TCID_50_ method. Samples were assayed in triplicate.

### ELISA and Western blotting

An enzyme-linked immunosorbent assay (ELISA) for human endostatin was performed with the Quantikine human endostatin immunoassay per the manufacturer’s protocol (R&D Systems, Minneapolis, MN). Conditioned media for the assay was obtained by seeding 5 × 10^5^ D283med or 7.5x10^5^ D425med cells in 6-well plates and infecting them the following day with MOI 0.1 MV-hEndo:Angio in a total volume of 500 μl OptiMEM. Unabsorbed virus was removed after two hours and the cells were incubated in 700 μl DMEM for an additional 48 hours. Infected cell supernatants were collected, centrifuged briefly, and then subjected to UV light exposure for 10 minutes to inactivate any residual virus. Samples were diluted 1:30 in assay diluent and run in triplicate. Data are presented as nanograms (ng) endostatin per ml per 10^4^ cells. Error bars represent +/− one standard deviation.

Western blotting was performed on conditioned media, prepared as described above, collected from D283med and D425 cells infected with either MV-hEndo:Angio or MV-mEndo:Angio. Briefly, 25 μl of D283med and D425med supernatants were resolved on a 10% SDS-PAGE gel and transferred to a PVDF membrane. After blocking, the MV-hEndo:Angio membranes were probed with a 1:1000 dilution of anti-angiostatin antibody (BAF226, R&D Systems) overnight, while the MV-mEndo:Angio membranes were probed with a 1:1000 dilution of anti-angiostatin antibody (PA1-600, Thermo Fisher Scientific, Rockford, IL) overnight. The following day the membranes were developed with Pierce ECL Western Blotting Substrate (Thermo Fisher Scientific).

### Production of conditioned media

Conditioned media for the HUVEC and bEnd.3 mouse endothelial cell studies was obtained by infecting semi-confluent 15 cm plates of Vero cells with MV-GFP, MV-hEndo:Angio, or MV-mEndo:Angio at an MOI of 0.01 in 5 ml total volume OptiMEM. After two hours, the OptiMEM containing virus was removed and replaced with 15 ml of DMEM + 10% FBS and the infected Vero cells were incubated at 37°C for an additional 48 hours. The media covering these cells was collected, centrifuged, aliquoted and stored at −80°C. Total protein concentration in the conditioned media was determined by Bradford assay (Bio-Rad, Hercules, CA). Residual virus was inactivated by exposure to UV light for 10 minutes prior to use.

### Endothelial cell tube formation and viability assays

Endothelial tube formation was evaluated with the Endothelial Tube Formation Assay (CBA200, Cell Biolabs Inc., San Diego, CA, USA). The supplied extracellular matrix (ECM) gel was thawed at 4°C and mixed to homogeneity using cooled pipette tips. A thin layer of ECM was then pipetted into the wells of a 96-well plate (50 μl/well) and allowed to polymerize at 37°C for 60 minutes. Two-3 × 10^4^ HUVECs or bEnd.3 stimulated with VEGF (10 ng/ml human VEGF or 20 ng/ml mouse VEGF) in 150 μl medium were added to each well on the solidified ECM gel. Culture medium was then added to each well in the presence or absence of MV-infected Vero conditioned media (10 μg/ml total protein concentration). The plates were incubated at 37°C for 18 hours and the endothelial tubes were observed using a fluorescent microscope after staining with Calcein AM dye. Three microscope fields were selected at random and photographed. Tube forming ability was quantified by counting the total number of cell clusters and branches under a 4X objective and four different fields per well. The results are expressed as mean fold change of branching compared with the control groups.

For viability/proliferation assays, HUVEC and bEnd.3 cells were seeded on 6-well plates at a density of approximately 1 × 10^5^ cells/well in M200 medium. Cells were treated with 10 μg/ml of MV-conditioned media one day after seeding. After two days, Alamar Blue reagent (Invitrogen) was added directly into culture media at a final concentration of 10% and the plates were incubated at 37°C. Optical density was measured spectrophotometrically at 540 and 630 nm three hours later. As a negative control, Alamar Blue was added to medium without cells. Each experiment was performed a minimum of three times using endothelial cells between passages three and eight.

### Migration assays

HUVEC and bEnd.3 migration was monitored using the wound-healing assay described by Thaloor et al. [31]. In brief, 3 × 10^4^ cells/well/ml were seeded in 24-well plates in M200 medium supplemented with low serum growth supplement (Cascade Biologics Inc.). After the cells had attached and formed a complete monolayer, a wound was made by scraping the surface of each well with a pipet tip. The cells were subsequently washed with PBS and incubated with the medium containing VEGF (10 ng/ml for HUVEC and 20 ng/ml for bEnd.3) with or without MV conditioned media (10 μg/ml). The width of the scraped area was photographed at different time intervals (0 and 18 hours) with a microscopic camera system (Leitz Diavert microscope, Leica, Bensheim; AxioCam, Carl Zeiss, Gottingen, Germany) at 40× magnification.

For quantitative analysis, HUVEC and bEnd.3 were grown in M200 containing low serum growth supplements until 40–50% confluent. Cells were washed with PBS, trypsinized, collected with 0.2% FBS and centrifuged at 300× g for 5 min. Cells were then resuspended with 0.2% FBS and counted using a Beckman Coulter Z2. A volume of 400 μl of this mix containing 5 × 10^5^ cells was placed on to Boyden Chambers (8 μm pore) inserts with and without MV conditioned media (10 μg/ml) in 24 well plates with 500 μl of M200. Human or mouse VEGF in 1% BSA was added to a final concentration of 10 or 20 ng/ml in the lower chambers as a chemo-attractant. The cells were then incubated at 37°C for 18–24 hrs. The Boyden chamber porous membranes were then blotted and fixed with 3.7% formaldehyde containing 0.05% crystal violet for 30 min. After repeated washes with distilled water, the membranes were air-dried. The migrated cells on the bottom side of the membranes were collected by scraping the bottom of the chamber with a Q-tip, which was subsequently placed into a 1.5 ml eppendorf tube and incubated in 80% methanol to extract the dye. The cells that remained on top of the membrane and within the Boyden chamber were separately incubated in 80% methanol, shaken at 500 rpm for 30 min, and the extracted dye measured at 570 nm. Migration was quantified using the ratio of the migrated cells over the total cells (migrated plus remaining cells) to determine the fraction of migrating cells in each individual experiment. Experiments were performed in duplicate.

### In vivo xenograft studies

The establishment of localized medulloblastoma tumors was conducted as previously described [7]. In brief, 5 ×10^5^ D283med-luc or 2.5x10^5^ D425med-luc cells suspended in 2 μl PBS were implanted into the caudate nuclei of 5–6 week-old Hsd:Athymic Nude-Foxn1nu mice (Harlan Laboratories, Indianapolis, IN). Bioluminescent imaging was conducted using the Xenogen Ivis Spectrum (Caliper Life Sciences, Hopkinton, MA) to ensure that the animals had roughly equivalent tumor burdens prior to being separated into treatment groups. These mice were subsequently treated with an intratumoral injection of the specified MV (2x10^5^ pfu/dose) or an equivalent volume of an OptiMEM vehicle control at the times outlined in the text. The animals were observed over the following weeks and euthanized if they became lethargic, displayed cachexia or exhibited hemiparesis or other motor impairment. All studies involving animals were approved by the Institutional Animal Care and Use Committee at The Research Institute at Nationwide Childrens’ Hospital.

At the time of necropsy, the brains were removed and fixed overnight in 10% buffered formalin phosphate. They were then paraffin embedded, cut into 4 μm tissue sections, and stained with hematoxylin and eosin (H&E). Individual sections were visualized under a Zeiss Axioskop 2 Plus microscope and photographed with a Zeiss AxioCam MRc camera (Carl Zeiss MicroImaging, LLC., Thornwood, NY).

### Human angiogenesis protein array

Proteome Profiler Human Angiogenesis Array Kits (R&D Systems, Minneapolis, MN) were used per the manufacturer’s instructions to detect the relative expression levels of 55 angiogenesis-related proteins in conditioned media-treated HUVECs and MV-treated mice bearing intracranial D283med-luc tumors. For HUVEC studies, whole cell lysate was made from HUVEC cells treated with 100 μg/ml MV-GFP or MV-hE:A conditioned media for 24 hours. After blocking the membranes, 300 μg of protein from the samples were added and incubated overnight at 4°C. The membranes were washed the next day and streptavidin-HRP was added or 30 minutes. Immunoreactive signals were visualized using Super Signal Chemiluminiscence substrate (Pierce) and Biomax MR and XAR film (Eastman Kodak Co.). Array data on developed X-ray film was quantified by scanning the film using Biorad Molecular Image Gel Doc™ XR + and analyzed using Image Lab™ software. Arrays for the *in vivo* studies were conducted in a similar fashion, using 300 μg lysate derived from excised D283med-luc tumors three days following MV treatment. Two tumors were analyzed for each treatment group.

### Dynamic contrast magnetic resonance imaging

T2-weighted imaging was performed 1 day pre- and 3, 7, 13, 20, and 27 days post treatment. DCE-MRI was performed 1 day pre- and 3 days post-treatment. The imaging was performed using a Bruker Biospin 94/30 magnet (Bruker Biospin, MA), a 2.0 cm diameter receive-only mouse brain coil, and a 70 mm diameter linear volume coil. T2-weighted images were collected using a T2-weighted RARE sequence (TR/TE = 3500/36 ms, RARE factor = 8, FOV = 20 × 20 mm2, matrix size = 256 × 256, slice thickness = 1 mm, navg = 1).

### Immunohistochemistry

Immunohistochemistry (IHC) was performed on paraffin-embedded tissues. IHC of tissue slides with anti-Measles Nucleoprotein antibody (NB100-1856; Novus Biologicals, Littleton, CO) was carried out as described previously [8]. Immunostaining for endostatin expression was carried out using anti-Endostatin antibody (1:50; NB100-91750, Novus Biologicals). CD31 expression was analyzed using anti-CD31 antibody (1:200; ECM590, Millipore, Billerica, MA). The number of cells staining positive for CD31 expression were counted by a blinded observer in 5 random 40× fields and treated versus controls compared (Student t test). Images were obtained with an Olympus AX70 fluorescence microscope and Spot v2.2.2 (Diagnostic Instruments, Sterling Heights, MI) digital imaging system.

### Statistical analysis

Survival curves were generated using the Kaplan-Meier method and GraphPad Prism version 5.01 software (GraphPad Software, Inc.). Comparisons of survival were done via the log-rank test. Differences were considered statistically significant if p ≤ 0.05. All other statistical analysis was performed using Microsoft Office Excel 2010 in Data Analysis using Regression or Student’s t test: paired 2-sample for means. Probabilities for the Student’s t test are listed as “P(T ≤ t) 2-tail” with an α of 0.05.

## Results

### Construction and oncolytic activity of measles viruses expressing endostatin:angiostatin fusion proteins

**Figure 1:**
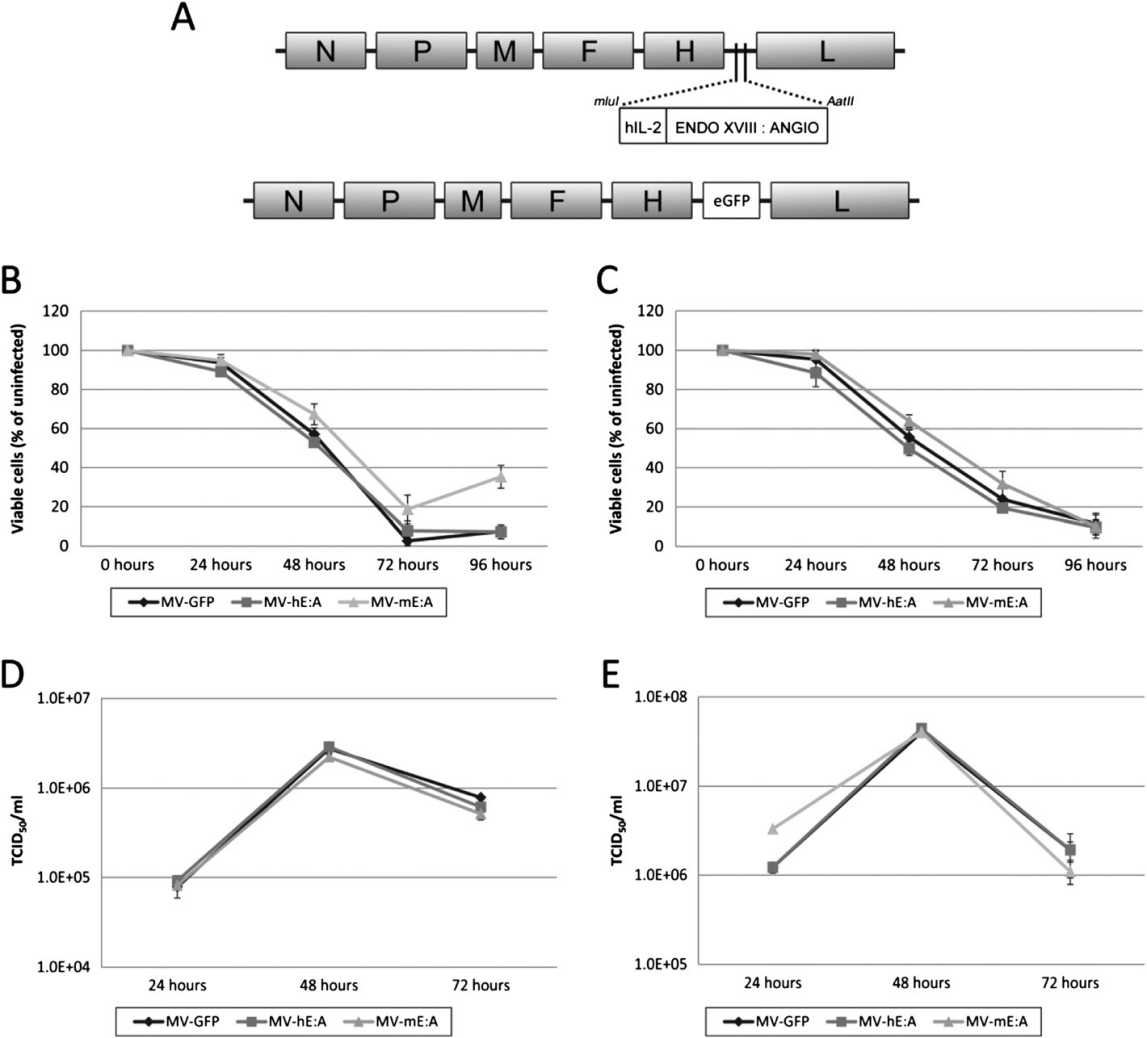
**Construction of MV-E:A viruses and evaluation of their cytopathic activity. (A)** Human/mouse E:A or enhanced GFP were cloned into the mluI/AatII restriction site of MV-NIS to create the MV-hE:A, MV-mE:A and MV-GFP viruses. The human IL-2 signaling peptide (hIL-2) appended to the E:A proteins results in their secretion from the infected cells. The oncolytic activity of these new viruses was compared by infecting **(B)** D283med and **(C)** D425med cells at an MOI of 0.1 and measuring their viability over the next three days by MTT assay. Viral production assays were similarly conducted by infecting **(D)** D283med and **(E)** D425med at MOI 0.1 and analyzing cell lysates collected at the listed timepoints. Viral titers were determined by the TCID_50_ method.

Human and mouse variants of an E:A fusion protein appended to the human Interleukin-2 signal peptide were cloned into the mluI/AatII restriction site of the parental MV-NIS virus (Figure 1A). The resulting viruses, designated MV-hE:A and MV-mE:A, were subsequently rescued as described elsewhere [29]. Since the insertion and location of an additional transcription unit in the MV genome can affect virus production, an MV encoding GFP at this position (MV-GFP) was also designed and rescued to serve as a control. We compared the oncolytic activity of these viruses *in vitro* by infecting the D283med and D425med medulloblastoma cell lines at MOI 0.1 and found the efficacy of the viruses to be roughly equivalent (Figure 1B-C). *In vitro* virus replication assays also showed that MV-hE:A, MV-mE:A and MV-GFP had similar growth kinetics (Figure 1D-E).

### Verification of endostatin:angiostatin production and biological activity

**Figure 2:**
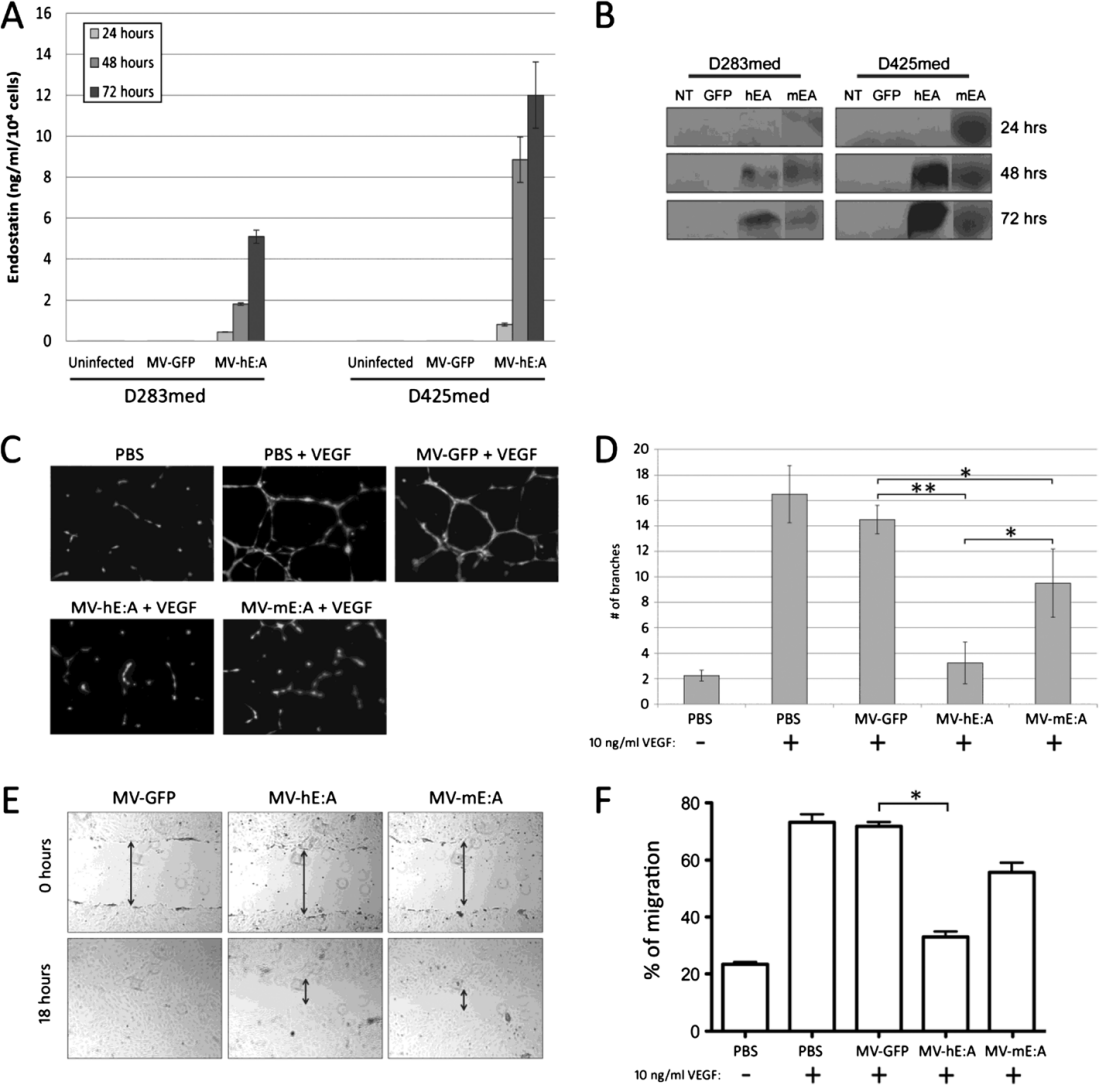
**MV-hE:A and MV-mE:A infection results in the secretion of active endostatin:angiostatin. (A)** Human endostatin production in infected D283med and D425med cells as quantified by ELISA. Endostatin concentration is expressed in ng/ml per 10^4^ cells. **(B)** Western blot analysis of media taken from either MV-hE:A or MV-mE:A infected D283med and D425med probed with an anti-angiostatin antibody. **(C-D)** Conditioned media from MV-hE:A and MV-mE:A infected Vero cells (10 μg/ml total protein) inhibits VEGF-mediated tube formation in HUVEC cells (*p ≤ 0.05, ** p ≤ 0.001). **(E)** To examine the effect of MV-E:A conditioned media on migration, scratch assays were performed in HUVECs by allowing the cells to move to the scraped region for 18 hours using VEGF (10 ng/ml) as a positive control in the presence and absence of MV conditioned media (original magnification × 40). **(F)** A crystal violet assay was also performed as described in Materials and Methods section to quantify the effect of MV conditioned media on migration (*p ≤ 0.05).

In order to determine if MV-hE:A and MV-mE:A infection induced medulloblastoma cells to secrete E:A, we performed ELISA and immunoblot analysis with D283med and D425med cell culture supernatants. Cells infected with MV-hE:A produced detectable quantities of endostatin, which increased steadily over 24–72 hours following infection as quantified by ELISA (Figure 2A). Supernatants from both MV-hE:A and MV-mE:A infected cells were then evaluated for angiostatin production, which presumably should be equal to endostatin production. Angiostatin expression from both MVs in both cell types was confirmed by an immunoblot with an anti-angiostatin antibody (Figure 2B).

We then performed a series of experiments to determine if the E:A being produced by the MV infected cells retained their biological activities. Tube formation assays were conducted by stimulating HUVECs with 10 ng/ml recombinant human VEGF in the presence or absence of MV-GFP, MV-hE:A and MV-mE:A conditioned media (10 μg/ml total protein). These conditioned media had previously been subjected to UV light in order to inactivate any residual infectious virus. Tube formation was evident in the PBS and MV-GFP treated samples within 24 hours, but was inhibited in the samples treated with MV-hE:A and MV-mE:A conditioned media (Figure 2C). Quantification of branch numbers showed that both MV-hE:A and MV-mE:A had a significant impact on tube formation compared to MV-GFP (p < 0.001 and p < 0.05 respectively), and that MV-hE:A had a greater effect in this regard over MV-mE:A (p < 0.05) (Figure 2D). Scratch assays were then performed in HUVEC monolayers to gauge the effect of the various MV conditioned medias on endothelial cell migration. Within 18 hours, the HUVECs had completely filled the void left in the MV-GFP samples, but evidence of the wounds was still visible in the samples treated with MV-hE:A and MV-mE:A (Figure 2E). A quantitative migration assay using the crystal violet method and 10 ng/ml human VEGF as a chemoattractant also showed that conditioned media from MV-hE:A and MV-mE:A inhibited endothelial cell migration whereas MV-GFP media had no effect over the PBS control samples (Figure 2F).

**Figure 3:**
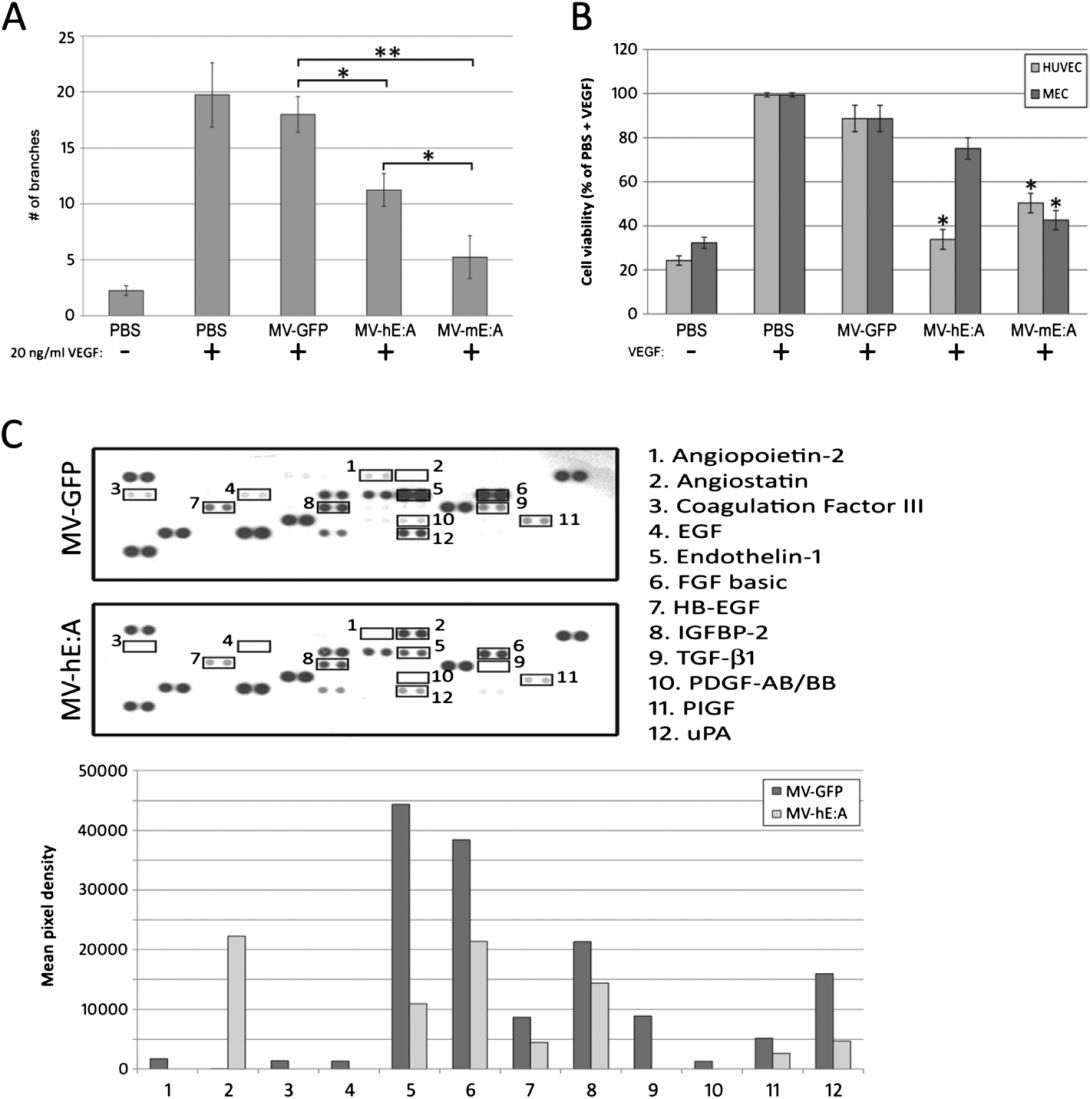
**MV-hE:A and MV-mE:A conditioned media inhibit angiogenic processes. (A)** Conditioned media (10 μg/ml total protein) from the MV-E:A viruses, but not MV-GFP inhibits tube formation in bEnd.3 mouse endothelial cells stimulated with 20 ng/ml recombinant mouse VEGF (*p ≤ 0.05, ** p ≤ 0.001). **(B)** MV-E:A conditioned media suppresses VEGF enhancement of cell viability in HUVEC and bEnd.3 cells (*p ≤ 0.05). **(C)** An angiogenesis protein array reveals that MV-hE:A conditioned media (100 μg/ml total protein) downregulates multiple angiogenic proteins in HUVECs compared to MV-GFP. Changes in protein expression are quantified in the accompanying bar graph.

Since the majority of new blood vessels formed in our medulloblastoma xenograft models would ostensibly be of murine origin, we also examined the effects of MV conditioned media on bEnd.3 mouse endothelial cells (MEC). We observed a significant decrease in VEGF-mediated MEC tube formation in samples treated with 10 μg/ml of MV-hE:A or MV-mE:A conditioned media relative to MV-GFP and PBS treated samples (Figure 3A). In contrast to the HUVEC tube formation assay where MV-hE:A was more effective in inhibiting tube formation (Figure 2D), MV-mE:A was significantly more effective at inhibiting MEC tube formation (p < 0.05). We next conducted viability assays with HUVEC and MEC cells stimulated by VEGF (10 ng/ml human VEGF and 20 ng/ml mouse VEGF respectively) and treated with 10 μg/ml of MV conditioned media or an equal volume of PBS. While the addition of VEGF led to increased cell proliferation irrespective of other treatment, MV-hE:A and MV-mE:A conditioned media were able to impede this process to some degree, each demonstrating superior activity against the endothelial cells of their native species (Figure 3B). We conducted similar experiments with the D283med and D425med medulloblastoma cell lines to determine if the MV conditioned media had a direct impact on their viability as well, but we observed no variance in cell viability even with concentrations of conditioned media up to 500 μg/ml (data not shown).

### Endostatin: Angiostatin inhibits angiogenic factors and blood vessel formation

In order to investigate whether the E:A produced by MV infected cells could inhibit angiogenic factors, we examined the levels of 55 proteins related to angiogenesis using a commercially available protein array (R&D Systems). Since the antibodies employed by this array were human-specific, we limited our focus to HUVEC treated with MV-hE:A and MV-GFP conditioned media. Relative to MV-GFP, MV-hE:A treatment resulted in decreased expression of angiogenic proteins such as angiopoietin-2, coagulation factor III, epidermal growth factor (EGF), endothelin-1, fibroblast growth factor (FGF), heparin-binding EGF-like growth factor (HB-EGF), insulin-like growth factor-binding protein 2 (IGFBP-2), transforming growth factor (TGF)-β1, platelet-derived growth factor (PDGF), placental growth factor (PlGF), and urokinase plasminogen activator (uPA) (Figure 3C).

**Figure 4:**
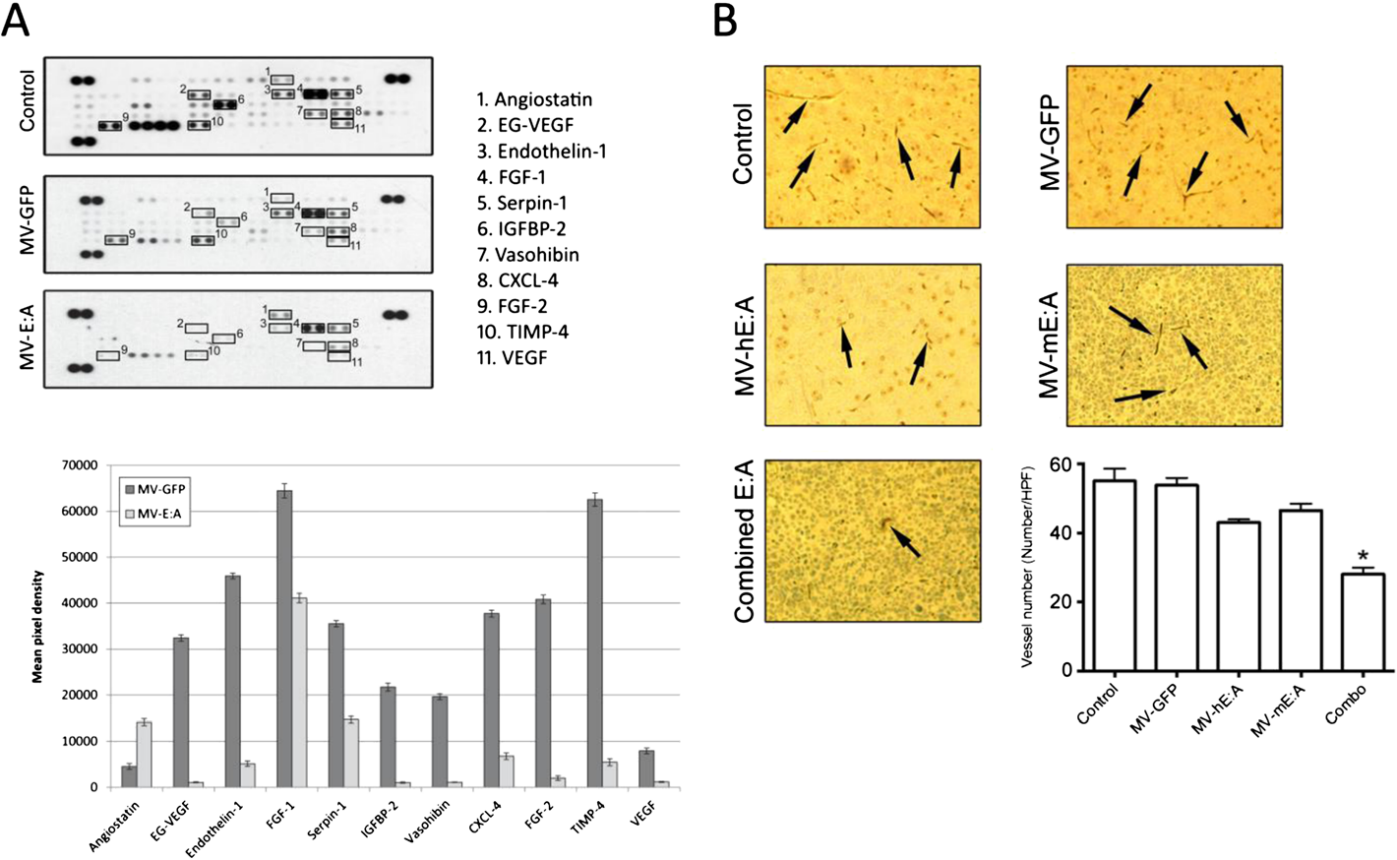
**MV-E:A infection downregulates multiple angiogenic factors and inhibits blood vessel formation in D283med-luc xenografts. (A)** Changes in angiogenic protein expression were monitored using a Proteome profiler antibody array as described in the Materials and Methods section. **(B)** IHC of representative tumors stained with an anti-CD31 antibody. The bar graph below displays the mean blood vessel counts of five randomly selected fields from each treatment group (*p < 0.05).

To examine whether MV-hE:A and MV-mE:A infection could similarly inhibit angiogenic factors in medulloblastoma tumors, we implanted 5 × 10^5^ D283med-luc cells into the caudate nuclei of athymic nude mice and treated them 30 days afterwards with a 2 × 10^5^ TCID_50_ dose of MV-GFP or a combined dose of 1 × 10^5^ TCID_50_ MV-hE:A and 1x10^5^ TCID_50_ MV-mE:A. The rationale for this combined MV-E:A approach was based on recent observations made in a xenograft model of glioblastoma wherein a significant portion of the vascular epithelium was found to be of neoplastic and thus human origin [32]. The animals were sacrificed three days after treatment and their tumors were carefully excised. Analysis of these tumor lysates revealed that the combined MV-EA treatment resulted in significant down-regulation of several angiogenic factors compared to the tumors treated with MV-GFP (Figure 4A). To assess the potency of single virus treatment in comparison to the treatment involving a combination of both viruses, D283med-luc tumors treated 7 days prior with 2x10^5^ TCID_50_ of a single virus (MV-hE:A or MV-mE:A) or 1x10^5^ TCID_50_ of each virus, were evaluated for blood vessel formation. While treatment with a single virus decreased blood vessel formation compared to MV-GFP treatment, only treatment with a combination of both viruses significantly decreased (p < 0.05) blood vessel formation, as revealed by IHC with an anti-CD31 antibody (Figure 4B).

### MV-E: A viruses prolong survival in mouse models of localized medulloblastoma

**Figure 5:**
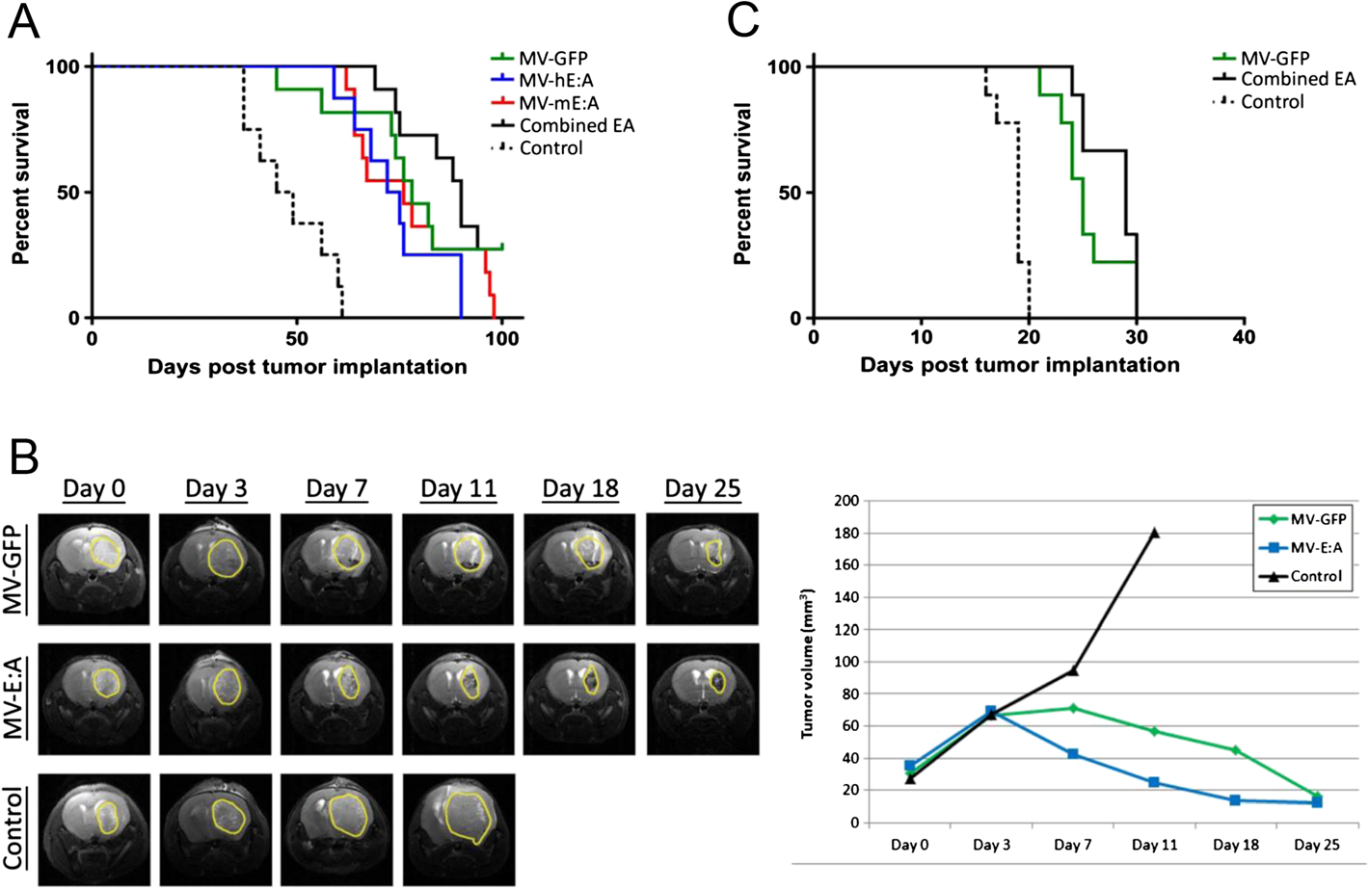
**MV-E:A viruses prolong survival in mouse xenograft models of medulloblastoma. (A)** Kaplan-Meier survival anaysis of mice implanted with D283med-luc treated 14 days later with a single intratumoral injection of the listed MVs (2x10^5^ TCID_50_). **(B)** Magnetic resonance imaging of representative mice from panel A. **(C)** Kaplan-Meier survival analysis of mice implanted with D425med-luc.

A mouse xenograft model of localized medulloblastoma was utilized to assess the efficacy of the MV-E:A viruses in prolonging survival [7]. Using stereotactic guidance, we implanted a total of 80 mice with 1 × 10^6^ D283med-luc cells. Bioluminescent imaging was performed 14 days later in order to verify that the tumors had properly established and were of roughly equivalent size on the basis of total emitted flux; animals displaying tumor dissemination or bioluminescent signals that fell outside of a standard deviation were excluded from further analysis. The remaining animals were placed into the following treatment groups: MV-GFP (n = 11); MV-hE:A (n = 8); MV-mE:A (n = 11), a combination of the MV-E:A viruses (n = 11), and a vehicle control (n = 8). We then treated the animals with a 2 × 10^5^ TCID_50_ intratumoral dose of their respective virus (combined MV-E:A animals received a 1 × 10^5^ TCID_50_ dose of both MV-hE:A and MV-mE:A) or an equivalent volume of optiMEM. The animals in the treated groups all displayed a significant prolongation in survival over the vehicle controls (p ≤ 0.0001), however the MV-hE:A and MV-mE:A viruses showed no benefit compared MV-GFP (Figure 5A). The combined MV-E:A treated animals showed a trend towards increased survival over MV-GFP (median survival times of 90 days versus 78 days), but this difference was not statistically significant. We also performed dynamic contrast-enhanced MRI with a subset of the control, MV-GFP and combined MV-E:A treated animals to make longitudinal assessments of individual tumors. Combined MV-E:A treated tumors appeared to regress more rapidly at first, but MV-GFP was able to produce a similar end result by 25 days after treatment (Figure 5B).

We performed a similar survival study with the D425med-luc line. Because D425med-luc in our experience grows more rapidly and generates more aggressive tumors, only 2.5x10^5^ cells were implanted in these mice. The treatment groups were as follows: MV-GFP (n = 9); the combined MV-E:A viruses (n = 9); and a vehicle control (n = 9). MV treatment of these tumors led to prolonged survival in the treated groups (p ≤ 0.0001), but there was again no significant benefit in using the combined MV-E:A viruses over MV-GFP (median survival times of 29 days versus 25 days) (Figure 5C).

**Figure 6:**
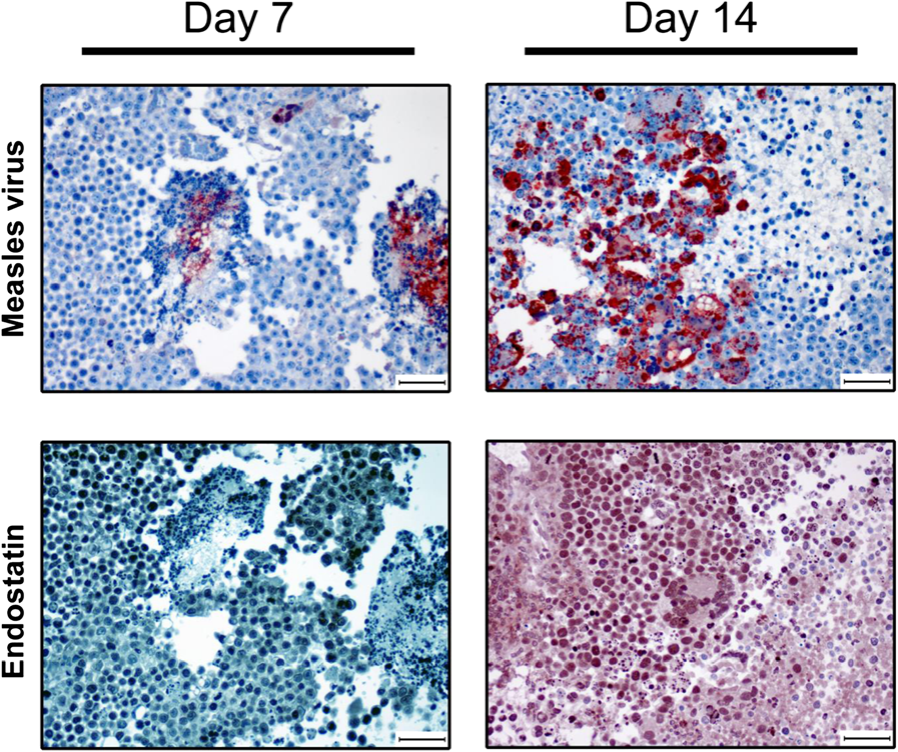
**Immunohistochemical Measles Virus and endostatin detection.** Paraffin embedded tissue sections derived from D283med-luc xenografts treated with MV-E:A 7 and 14 days prior, were stained with rabbit polyclonal MV nucleoprotein and endostatin antibodies. Nucleoprotein immunoreactivity was detected in the cytoplasm of individual cells and multi-nucleated syncytia at both days evaluated. Positive endostatin immunostaining was observed surrounding MV nucleoprotein expression. Scale bars represent 50 microns.

In an attempt to explain why survival was not enhanced with E:A expression, D283med-luc tumors were evaluated 7 and 14 days following MV-E:A for MV and endostatin expression (Figure 6). Immunostaining for MV nucleocapsid protein was positive at both time points. Serial sections revealed positive endostatin staining surrounding MV replication. However, active MV replication and concomitant endostatin expression was very sparse within the tumor.

## Discussion

Although there are more than 100 subcategories of brain tumors with different biological characteristics, each is reliant on the generation of new blood vessels for survival and growth providing a rationale for the inclusion of anti-angiogenic agents in their treatment [17]. The use of such therapies in the treatment of medulloblastoma has thus far been surprisingly limited, but recent case studies have demonstrated improved progression-free survival in patients treated with the anti-VEGF monoclonal antibody Bevacizumab in combination with other chemotherapeutics [33,34]. Aside from VEGF, medulloblastomas have been shown to produce several factors that contribute to angiogenesis including basic FGF, angiopoetin-1 and −2, TGF-α, and PDGF-A [35]. As such, prospective anti-angiogenesis therapeutic strategies that target only a single angiogenic factor or pathway could ultimately prove to be inadequate.

Endostatin and angiostatin are two endogenous and broad-spectrum inhibitors of angiogenesis. While numerous studies have demonstrated impressive anti-angiogenic and antitumor activities with these agents in rodent models, similar findings have not materialized in phase I/II trials with human patients [36-39]. Several factors have hindered the advancement of endostatin- and angiostatin-based therapies, such as short serum half-lives, manufacturing difficulties, and issues pertaining to their solubility and stability [25,26,40]. Endostatin and angiostatin are also not directly cytotoxic to the tumor cells themselves and instead must be continually delivered to the tumor microenvironment in order to inhibit angiogenesis [41]. In this study, we developed oncolytic MVs that encode human or mouse variants of E:A fusion proteins, which display enhanced anti-angiogenic activity and prolonged half-lives compared to endostatin and angiostatin expressed individually [42]. Moreover, their incorporation into the genome of a replication competent oncolytic virus assures their continued expression as long as the virus is able to infect and replicate in susceptible cells.

The MV-hE:A and MV-mE:A viruses are derivatives of MV-Edm, a highly attenuated vaccine strain with an excellent safety profile that extends more than 50 years and encompasses over a billion recipients worldwide [9]. In contrast to wild-type MV, which primarily uses the signaling lymphocyte activation molecule expressed by various lymphocytes as an entry receptor, MV-Edm has adapted to use the more ubiquitous membrane cofactor protein, also known as CD46 [43]. As a negative regulator of the complement system, CD46 is expressed by all nucleated cells in the human body. Despite such widespread distribution, CD46 expression levels on normal cells are generally low and fall under the threshold of receptor density required to initiate and sustain an MV-Edm infection [44]. Most tumors express elevated levels of CD46 however, and are consequently highly susceptible to MV-Edm oncolysis [7,45-48]. The reliance of MV-Edm on CD46 receptor density allows the virus and its derivatives to discriminate between tumor and normal cells, infecting and lysing the former while sparing the latter. Phase I clinical trials have demonstrated the safety of these viruses for the treatment ovarian cancer and glioblastoma, where no dose-limiting toxicity has been observed following administration of the MV at doses up to 10^9^ TCID_50_ delivered intraperiotoneally and 10^7^ TCID_50_ for MV delivered through the central nervous system respectively [6,49].

We reasoned that a recombinant MV-Edm would be an optimal vector to deliver anti-angiogenic agents because of its oncolytic activity, overall safety, and specificity for infecting and replicating in tumor cells. The addition of E:A fusion genes to the MV-Edm genome did not attenuate the viruses’ cytotoxicity or replication in the D283med or D425med medulloblastoma cell lines (Figure 1). Moreover, we were able to verify that the E:A being expressed by the infected cells was physiologically active. Conditioned media derived from MV-E:A infected cells inhibited viability in activated endothelial cells and impeded their migration and formation into tube-like structures (Figures 2 and 3). In vivo, a single low-dose injection of MV-E:A delivered intratumorally resulted in the down-regulation of multiple angiogenic modulators within three days (Figure 4) and decrease blood vessel formation (Figure 4). Despite these initially promising observations, the MV-E:A viruses ultimately failed to significantly prolong survival in the mouse xenograft models of medulloblastoma over MV-GFP (Figure 5). Although we can surmise that E:A is being expressed by the infected tumors through our angiogenesis protein array (Figure 4) and IHC (Figure 6) data, it is very likely that the anti-angiogenic effect we witnessed early on dissipated over time, perhaps as pockets of tumor cells that escaped MV oncolysis continued to grow and initiate the processes of neovascularization without E:A to impede them. Evaluation of tumors for MV replication (Figure 6) supports this notion as there were only small foci of active replication within the tumor. If this is indeed the case, increasing the amount of virus administered and/or fractionating the dosing regimen to aid the spread of the virus could prove to be beneficial. Another possible reason for the lack of synergy may simply be due to inadequate production of the E:A transgenes. It is well established that the location of a transgene within the MV genome dictates its relative abundance, with genes closer to the 3′ end of the genome being transcribed and translated in greater quantity [50]. In the case of the MV-hE:A and MV-mE:A viruses, the E:A transgenes have been inserted between the measles H and L genes, near the 5′ end of the genome (Figure 1A). Cloning these genes into a site further upstream would result in higher expression of E:A, albeit at the expense of reduced virus titers. Further experimentation would be required to determine if this is an acceptable tradeoff.

Aside from the MV-E:A viruses described here, other oncolytic viruses armed with E:A fusion proteins have also recently been described in the literature. Yang and colleagues reported enhanced efficacy using the attenuated herpes simplex virus-1 mutant, G207, armed with human E:A for the treatment of lung cancer [51]. Xenograft flank tumors treated with 1 × 10^7^ pfu of the E:A armed virus were found to be consistently smaller than those treated with the parental G207 virus up to day 13 post treatment. The effects of this virus on overall survival, however, were not investigated. Tysome and colleagues have also reported enhanced efficacy and survival in mouse xenograft models of pancreatic cancer following treatment with a modified Lister strain of vaccinia virus [41]. Decreased microvessel density counts and reduced tumor burdens were also observed in the mice treated with the E:A expressing virus relative to the parental vaccinia strain. These results were achieved with two intratumoral dosing regimens: a low dose consisting of three separate injections of 1 × 10^7^ pfu virus and a high dose consisting of six injections of 5 × 10^7^ pfu virus. An intravenous delivery method was also examined, but it was terminated due to excessive toxicity before any efficacy could be observed. It is difficult to make direct comparisons between these reports and our own because of the vast biological differences of the viruses and tumors under study. It appears that the inclusion of E:A in oncolytic virotherapy can have the potential to be beneficial in some circumstances, but there is still considerable room for further optimization and improvement. Further modifications with the MV-E:A viruses, such as altering their dosing regimen and levels of transgene expression, will hopefully lead to superior oncolytic measles virotherapy for the treatment of medulloblastoma. However, there is the possibility that inclusion of E:A may not significantly increase the already significant oncolytic potential of MV.

## Conclusions

Our results show that the angiogenesis inhibitors, endostatin and angiostatin, expressed by a recombinant MV significantly reduced endothelial cell growth, viability, and migration. Treatment of our mouse model of medulloblastoma with MV-E:A decreased the number of tumor-associated blood vessels and prolonged the animal’s survival compared to control treated animals. However, the increased survival did not significantly improve survival compared to MV-GFP treated animals in this model. Measles therapy coupled with anti-angiogenesis therapy may have therapeutic benefit against medulloblastoma clinically.

## Competing interests

Authors declare that they have no competing interests.

## Authors’ contributions

BH participated in the conception and design, development of methodology, acquisition of data (all in vitro and in vivo studies), analysis and interpretation of data, and helped draft the manuscript. HKB was involved in the conception and design, development of methodology, acquisition of data (in vitro and in vivo angiogenesis studies), analysis and interpretation of data, and helped draft the manuscript. PJH was involved in the conception and design and interpretation of data. CRP carried out all histopathological review and provided interpretation of the disease via microscopic evaluation. KP supervised the dynamic contrast magnetic resonance imaging and provided interpretation of the results. AB performed the dynamic contrast magnetic resonance imaging. CR participated in the conception and design of the study. AWS was involved in the conception and design, development of methodology, animal studies, analysis and interpretation of data, helped draft the manuscript, and supervised the study. All authors read and approved the final manuscript.

## Pre-publication history

The pre-publication history for this paper can be accessed here:

http://www.biomedcentral.com/1471-2407/14/206/prepub
